# Novel Systems Modeling Methodology in Comparative Microbial Metabolomics: Identifying Key Enzymes and Metabolites Implicated in Autism Spectrum Disorders

**DOI:** 10.3390/ijms16048949

**Published:** 2015-04-22

**Authors:** Colin Heberling, Prasad Dhurjati

**Affiliations:** 1 Department of Biotechnology, Johns Hopkins University, Rockville, MD 20850, USA; 2 Department of Chemical and Biomolecular Engineering, University of Delaware, Newark, DE 19716, USA; E-Mail: dhurjati@udel.edu

**Keywords:** autism, metabolomics, comparative, microbial, computational, bioinformatics, biomarkers, gut, gastrointestinal, microbiome

## Abstract

Autism spectrum disorders are a group of mental illnesses highly correlated with gastrointestinal dysfunction. Recent studies have shown that there may be one or more microbial “fingerprints” in terms of the composition characterizing individuals with autism, which could be used for diagnostic purposes. This paper proposes a computational approach whereby metagenomes characteristic of “healthy” and autistic individuals are artificially constructed via genomic information, analyzed for the enzymes coded within, and then these enzymes are compared in detail. This is a text mining application. A custom-designed online application was built and used for the comparative metabolomics study and made publically available. Several of the enzyme-catalyzing reactions involved with the amino acid glutamate were curiously missing from the “autism” microbiome and were coded within almost every organism included in the “control” microbiome. Interestingly, there exists a leading hypothesis regarding autism and glutamate involving a neurological excitation/inhibition imbalance; but the association with this study is unclear. The results included data on the transsulfuration and transmethylation pathways, involved with oxidative stress, also of importance to autism. The results from this study are in alignment with leading hypotheses in the field, which is impressive, considering the purely *in silico* nature of this study. The present study provides new insight into the complex metabolic interactions underlying autism, and this novel methodology has potential to be useful for developing new hypotheses. However, limitations include sparse genome data availability and conflicting literature experimental data. We believe our software tool and methodology has potential for having great utility as data become more available, comprehensive and reliable.

## 1. Introduction

Autism spectrum disorders are a category of mental illnesses characterized by social cognitive impairments and stereotyped behaviors [1]. Autistic individuals often have trouble fitting into society and put a financial burden on their families, lifelong. Autism diagnosis has been steadily on the rise in the past decade or so, affecting one in every 88 children according to a source in 2012 [1] and one in every 68 children from current data (2015) from the Centers for Disease Control and Protection [2]. There are most likely many adults suffering from autism that have never been diagnosed. This is because there is as of yet no clear method to diagnose autism; diagnosis is purely based on making qualitative observations of an individual. This is why there is a pressing need to find reliable methods for autism diagnosis and for treatment, as well.

Recent research suggests that individuals with autism often suffer from gastrointestinal dysfunction, as well [3,4]. Rather than focusing on the illness by way of human genetics, many scientists are now exploring the impact of microbial genetics. There exists a full isolated ecosystem of microbial lifeforms that inhabit the human gastrointestinal tract. These microbes have a profound impact on the health and disease of the human host and in general have a symbiotic co-existence with the host [5]. Microbial species or strain composition is believed to largely contribute to homeostasis or abnormality in humans. While each person’s microbial “fingerprint” is unique, there are specific patterns seen in those that are healthy and those that have specific illnesses [6,7]. Remarkably, autism has been correlated with the overgrowth of certain types of bacteria, such as certain species of *Clostridia* [8,9] and *Desulfovibrio* [9,10], and there is some preliminary evidence that *Sutterella* may be implicated, as well [11]. Data from the pyrosequencing study by Finegold *et al.* [9] suggested that several other organisms may be implicated in autism, as well, but these results were less significant than those implicating *Clostridia* and *Desulfovibrio*.

Nonetheless, one organism, *Akkermansia*, was chosen from among these organisms to be included in the study, to see how much of an impact these less significant organisms might have. *Akkermansia* is part of the *Verrucomicrobia* phylum. According to a study by Williams *et al.* [12] where live intestinal biopsies were taken to measure bacterial composition, *Verrucomicrobia* made up approximately 1% of the total bacterial composition in individuals with autism and only 0.5% in healthy age-matched controls. This was another leading factor for the decision to include *Akkermansia* in the present study.

As one might expect, as bacterial composition has an effect on health and disease, the molecular metabolites that these microbes produce are the “tools” that carry out these biological changes. For example, individuals with autism have been cited as having sulfur metabolic deficiencies, to suffer from elevated oxidative stress and to have trouble detoxifying xenobiotic compounds and heavy metals [13,14,15]. It is therefore important to analyze the metabolome of these individuals and that of healthy individuals (for control) in order to identify useful biomarkers and perhaps even gain a greater understanding of the disorder. Knowledge may be gained about other related illnesses, as well, such as others related to gastrointestinal dysfunction.

Metagenomics is the study of the genetic content contained in whole microbial ecosystems isolated from natural environments [16,17]. In contrast, the more traditional genomics is the study of only DNA from a single, isolated species or strain of organism. Metagenomics therefore brings with it new challenges in bioinformatics. This is because before regular genomics can be applied to each organism, the key organisms need to be identified by aligning sample DNA reads to reference sequences contained in curated databases [18]. Of course, the environment that this study focuses on is the human gut microbiome. This study does not use the conventional methods of metagenomics, but rather simplifies the problem by constructing artificial metagenomes based on genomes of organisms already known to be contained in the target environment. There are numerous pieces of literature already describing the composition of microbes contained in autistic individuals and “healthy” individuals. The present study constructs a metagenomics “model”, rather than conducting a pure metagenomics study.

## 2. Methods

The literature suggests that there are distinct microbiomes characteristic of a “healthy” individual and that of an autistic individual [4]. Therefore, the first step was to choose the microorganisms to represent these microbiomes. It was the intended goal to be as comprehensive as possible with this step, so that all possible enzymes and metabolites that are possible to exist in urinary or stool samples are covered. Choosing the correct microorganisms can be tricky, as sometimes there are conflicting data or interpretations of data in different literature [19]. Another quandary is whether to include the more common organisms in both microbiomes studied or to only include the “problem” organisms; that is, the microorganisms thought to cause the main differences between the two microbiomes. In the latter case, the autistic microbiome could be represented with just a few species of microbe. The end goal is to find the key enzymes and metabolites expressed that are entirely different in the autistic individual. Of course, autism is a complex disorder with multiple different pathways for pathogenesis, so the autistic microbiome chosen will try to represent all cases of autism arising from gut microbiological origin.

Only those microorganisms thought to be represented in significantly higher amounts (proportionally) in the guts of autistic individuals were chosen for the autistic microbiome, with the core microorganisms representative of a healthy human gut for the control microbiome. However, there is still no consensus on what defines a typical “healthy” gut microbiome, so the “control” microbiome was chosen based on a few key steps. First, those microbes that have been reported to be in decreased quantities in autism were included in the “control” microbiome [9,12,20]. Next, those reported to have no change in composition were included. Finally, other organisms that are normally found in the human gut, but that had very little discussion in related autism literature were included in the “control” microbiome. To facilitate this final step, we have unpublished work where the MEtaGenome Analyzer (MEGAN software) [21,22] was used to analyze three sets of metagenomic data from the Human Microbiome Project’s collection of human stool samples [6,23]. These design criteria may seem confusing at first if we take into consideration that those microorganisms that are decreased in autism are nonetheless still implicated in autism, just like those organisms that are found in increased amounts. However, our design considerations are predicated on the assumption that those organisms that are found in decreased numbers in autism are in fact usually beneficial for the host in otherwise “healthy” microbiomes. There is at least some evidence for two such cases: *Lactobacillus* species and *Bifidobacterium* species [24]. We can therefore justify stratifying gut microbes into two groupings based on a simple binary distinction: those microbes that are found in increased numbers in autism and those microbes that are not found in increased numbers in autism. Comparing these two groups should reveal key differences in expressed enzymes. Thus, finding enzymes that are expressed in the “autism” group and not in the “control” group may identify metabolites indicative only of our “problem” organisms and thus may serve as potential biomarkers for autism. On the other hand, finding enzymes that are expressed by the “control” group and not the “autism” group may also identify key biomarkers, except in this case, clinicians may be able to look for decreased quantities of such molecules instead.

Enzyme Commission (EC) numbers were used for the comparative analysis. EC numbers are a standardized way to identify enzymes, much like CAS numbers (Chemical Abstracts Service) for common chemicals. GenBank files for each microbe were downloaded from the National Center for Biotechnology Information (NCBI) [25]. The limitations of this study occurred at this stage. Not all microbes known have a completely sequenced genome uploaded to NCBI, and even less still have their protein products annotated with EC numbers. This greatly limited which organisms could be used in the study. The following list organizes microorganisms into three categories: those included in the first software query, those additional organisms that were able to be included in Query 2 after *de novo* EC number annotations were made (more detail later) and those organisms that were desired to be included, but in the end were not.

The usual approach for metagenomics presents significant bottlenecks for analysis and data storage. This is why the idea came about that there may be another approach that is more efficient in very specific circumstances. Instead of using metagenomic data, this study attempts to construct an artificial metagenome by carefully choosing the microorganisms to include in the model and using the curated database of GenBank files to analyze the full spectra of enzymes coded in the genomic DNA of these organisms. This study is differentiated from a proteomics study, because we are only interested in proteins that have been assigned a standardized Enzyme Commission (EC) number, so that there is no ambiguity between gene products. Two enzymes from two different organisms may have the same EC number, but slightly different amino acid sequences or slightly different protein names in the GenBank annotation files. Using EC numbers will allow automated programs to match identical enzymes correctly. The enzymes are correlated with their associated chemical reactions and the implicated metabolites. This method of intentionally leaving out some information and looking at the bigger picture on more of a systems level could be classified as systems biology [26].

**Figure 1:**
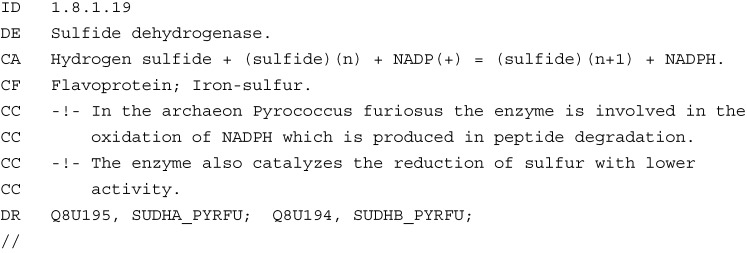
Example data extracted from The ENZYME Database in 2000, Bairoch [28].

A personal computer was used for all programming and analysis. A simple, personally-designed online application was used for all analysis. This software can be found at the web link [27]. Full instructions on how to use the software can be found in a link to the readme file on the main program interface linked above. The software utilizes a relational database system using MySQL to store and query information from the GenBank annotation files from NCBI and information from an EC number database [28]. The EC number data file was parsed for inserting data into a personally-designed database table. The following example (Figure 1) shows the type of information contained in the data file that was parsed for EC numbers.

The web application allows its user to choose two groups of microbiomes for metabolomics comparison. The program was designed with the intent to be used for any microbial metabolomics study between two microbiomes, two small alterations of the same microbiome or even simply between two single organisms. The application allows the user to upload a new genome file in GenBank format. Initially, the application could only accept GenBank files with EC number annotations present, but now, there does not need to be any EC number annotations in the original file. However, inserting EC number annotations with automation has certain limitations. Database queries fall into three categories for each comparison: no matches, one unique match or more than one match. In the event that there is more than one match, manual curation will be necessary. In the meantime, the program instead prints the first three matches to the GenBank file and makes a designation within the file and within the associated database that says that the information may be less reliable than unique matches or the data that was already annotated in the original file.

**Figure 2:**
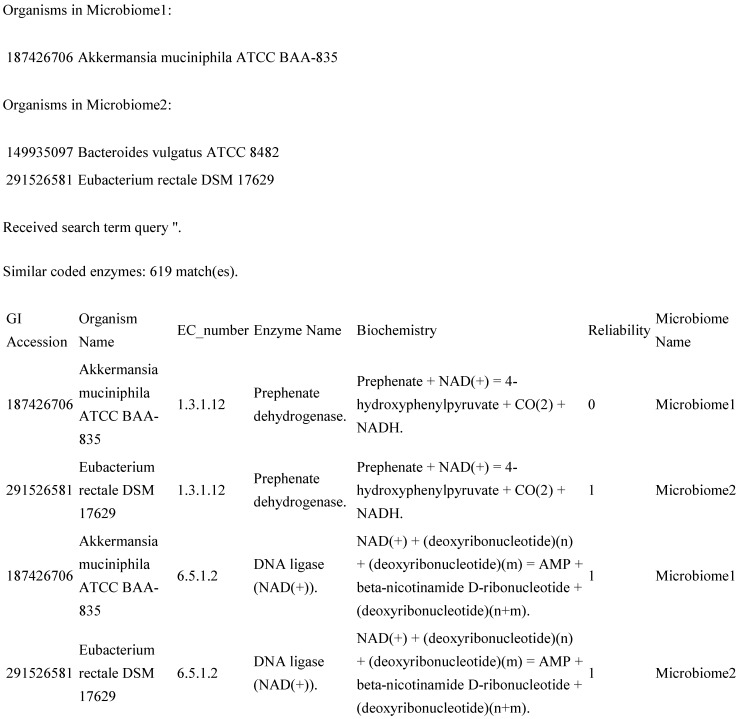
Example extract from the original software results page.

The application returns output directly to the screen within the web browser. The output consists of a data table, where each row contains columns showing the GI accession number (from NCBI) of the organism that the data come from, the organism’s common name, an EC number, the predominant name associated with that EC number, the associated biochemistry, a “reliability factor” and a keyword describing which microbiome the data are associated with (default “Microbiome 1” and “Microbiome 2”, or custom names supplied by the user on the form page). The table is broken up into three major sections: those enzymes that are identical between the two microbiomes and those enzymes that are unique for each microbiome. For this study (and likely others that might benefit from this software package), the sub-tables showing the unique results are most useful. An example partial result (Figure 2) might be as follows.

In Figure 2, GI accession is the unique identifier for that GenBank record within NCBI, and the reliability column differentiates between new annotations with multiple EC number matches (reliability = 0) and new annotations with unique matches and old annotations (reliability = 1).

Three queries total were made with the web application. The first involved the organisms from the top section of Table 1. The second added the five organisms from the middle section of Table 1. In the third query, we tried to filter our results to just the organisms that are thought to be most important in each microbiome. These microbiomes consisted of those in Table 2. Table 1 is also available in the Supplemental Material as Table S1.

**Table 1:** Microorganisms chosen for the comparative metabolomics study. Organisms included in Query 2 had *de novo* Enzyme Commission (EC) number annotations.

Query	“Healthy” Microbiome	“Autism” Microbiome
***Included in Query 1***	Bacteroides fragilis	Desulfovibrio desulfuricans
	Bifidobacterium longum	Clostridium perfringens
	Ruminococcus bromii	Clostridium difficile
	Roseburia intestinalis	Akkermansia muciniphila
	Faecalibacterium prausnitzii	
	Eubacterium rectale	
	Alistipes putredinis	
	Alistipes shahii	
	Weissella koreensis	
	Prevotella ruminicola	
	Odoribacter splanchnicus	
	Methanobrevibacter smithii	
	Clostridium leptum	
	Lactobacillus acidophilus	
***Additional organisms included*** ***in Query 2***	Bacteroides thetaiotaomicron	Sutterella wadsworthensis
	Bacteroides caccae	Clostridium bolteae
		Bacteroides vulgatus
***Desired, but not included***	Bacteroides finegoldii	Desulfovibrio piger
	Bacteroides ovatus	Desulfovibrio intestinalis
	Bacteroides uniformis	Clostridium butyricum
	Bifidobacterium adolescentis	Clostridium paraputrificum
	Bifidobacterium pseudolongum	Clostridium subterminale
	Eubacterium siraeum	Clostridium tertium
	Dorea	Clostridium bifermentans
	Veillonella	Clostridium glycolicum
	Turicibacter	Bacteroides stercoris
	Barnesiella intestinihominis	Parabacteroides distasonis
	Odoribacter laneus	Parabacteroides merdae
	Dialister invisus	Paraprevotella xylaniphila
		Eubacterium eligens
		Prevotella oulorum

**Table 2:** Selected organisms for Query 3 of the Metabolomics Software.

**Organisms in Control:**	
392623967	Bacteroides caccae CL03T12C61
60495220	Bacteroides fragilis ATCC 25285 = NCTC 93
29342100	Bacteroides thetaiotaomicron VPI-5482
666001751	Bifidobacterium longum BXY01
488447870	Lactobacillus acidophilus La-14
148552872	Methanobrevibacter smithii ATCC 35061; PS; DSMZ
**Organisms in Autism:**	
149935097	Bacteroides vulgatus ATCC 8482
480704622	Clostridium bolteae 90A9
110676061	Clostridium perfringens ATCC 13124
219869941	Desulfovibrio desulfuricans ATCC 27774
115252745	Peptoclostridium difficile 630
512689910	Sutterella wadsworthensis HGA0223

The organisms chosen for each query were input into the program and the results copied and pasted into an Excel file (available as the Supplemental Material, Tables S2–S7: each query is separated into two worksheets, one for complete raw data, and one for filtered data of interest). The “unique” results were looked through manually and considered on a case by case basis, with literature data in mind. Special attention was paid to those enzymes and metabolites found in the methionine and cysteine metabolic pathways and those involved with oxidative stress [13] and amino acid metabolism [29,30,31].

## 3. Results

The full results will not be reproduced here, but can easily be reproduced using the online software and the methods detailed above. The full results for each query are available as the Supplemental Material, Tables S2, S4, and S6. Additionally, the full results have been filtered for the most meaningful results and pasted within the Supplemental Material, Tables S3, S5, and S7. The first query resulted in the most meaningful results. It had 19,959 identical enzyme matches between the two microbiomes tested, 834 unique enzymes for the “control” microbiome and 161 unique enzymes for the “autism” microbiome. Gene copies are included in this count, so these numbers are a bit inflated. The identical enzymes are meaningless for this study, so we direct our attention to the tables of “unique” enzymes. Any meaningful results must be found manually. Table 3 below shows the differences in match statistics between each query. The majority of the results found with Query 1 were also found in Queries 2 and 3, with possibly a few minor differences. Judging by the quantity of the results, these minor differences were assumed to be near negligible. Therefore, when comparing Queries 1 and 2, we can come up with an approximate difference in the number of unique results by subtracting the statistics. Therefore, for instance, Query 2 only had 19 new results unique to the “control” microbiome and 34 new results unique to the “autism” microbiome, compared to the results of Query 1. Thus, one can see that Queries 2 and 3 would have less novel results overall than Query 1, given that Query 1 was conducted first.

**Table 3:** Comparing match statistics between web application queries.

Query	Similar Enzymes	Control Unique	Autism Unique
1	19,959	834	161
2	41,035	853	195
3	14,451	356	387

From an experimental study on metabolic biomarkers in autistic individuals [29], we know that many amino acids are found in significantly lower abundance *vs.* controls, including glycine, serine, threonine, alanine, histidine, glutamine, glutamate and the organic acid, taurine. Antioxidants (especially glutathione) are also in much lower abundance. Another study [30] cites reduced glutathione as being in much lower abundance and another type of antioxidant, thioredoxins, as being in much higher abundance. James *et al.* [13] claims that there is a metabolic bottleneck in many autistic individuals, where the conversion of methionine to cysteine is inhibited, leading to diminished glutathione formation. This information was kept in mind when analyzing the data. The relevance of these findings will be discussed in more detail in the Discussion Section.

In Table 4, Table 5, Table 6, Table 7, Table 8, Table 9, Table 10 and Table 11 we present the most meaningful results obtained from the data mining. The majority of the results come from the first web application query. First, for the enzymes unique to the “control” microbiome, we present enzymes indicative of glutamate metabolism. Some of the entries below contain notation, such as “×3” after an organism name. This denotes that that organism has that number of gene copies coding for that particular enzyme (the first table with this notation is Table 6 below).

**Table 4:** Acetylornithine transaminase expression (“control” unique).

2.6.1.11	Acetylornithine Transaminase	*N*(2)-Acetyl-l-ornithine + 2-Oxoglutarate = *N*-Acetyl-l-glutamate5-semialdehyde + l-Glutamate
**Expressed by the following organisms:**		
291516108	Alistipes shahii WAL 8301	
291516108	Alistipes shahii WAL 8301	
60495220	Bacteroides fragilis ATCC 25285 = NCTC 93	
291526581	Eubacterium rectale DSM 17629	
291526581	Eubacterium rectale DSM 17629	
295102938	Faecalibacterium prausnitzii L2/6	
148552872	Methanobrevibacter smithii ATCC 35061; PS; DSMZ	
324314063	Odoribacter splanchnicus DSM 220712	
294473972	Prevotella ruminicola Bryant 23	
291541371	Roseburia intestinalis XB6B4	

**Table 5:** Phosphoserine transaminase expression (“control” unique).

2.6.1.52	Phosphoserine Transaminase	(1) *O*-Phospho-l-serine + 2-Oxoglutarate = 3-Phosphonooxypyruvate + l-Glutamate (2) 4-Phosphonooxy-l-threonine + 2-Oxoglutarate = (3*R*)-3-Hydroxy-2-oxo-4-phosphonooxybutanoate + l-Glutamate
**Expressed by the following organisms:**		
167660682	Alistipes putredinis DSM 17216	
291516108	Alistipes shahii WAL 8301	
60495220	Bacteroides fragilis ATCC 25285 = NCTC 93	
295102938	Faecalibacterium prausnitzii L2/6	
324314063	Odoribacter splanchnicus DSM 220712	
294473972	Prevotella ruminicola Bryant 23	
291541371	Roseburia intestinalis XB6B4	
291543183	Ruminococcus bromii L2-63	

**Table 6:** 2-oxoglutarate synthase expression (“control” unique).

1.2.7.3	2-Oxoglutarate Synthase	2-Oxoglutarate + CoA + 2 Oxidized Ferredoxin = Succinyl-CoA + CO(2) +2 Reduced Ferredoxin + 2 H(+)
**Expressed by the following organisms (multiple gene copies present):**		
291516108	Alistipes shahii WAL 8301 (×5)	
148552872	Methanobrevibacter smithii ATCC 35061; PS; DSMZ (×5)	
324314063	Odoribacter splanchnicus DSM 220712 (×3)	

**Table 7:** Glutamate synthase expression (“control” unique).

1.4.1.14	Glutamate Synthase (NADH)		2 l-Glutamate + NAD(+) = l-Glutamine + 2-Oxoglutarate + NADH
**Expressed by the following organisms:**			
291526581		Eubacterium rectale DSM 17629 (×3)	
295102938		Faecalibacterium prausnitzii L2/6 (×4)	
291541371		Roseburia intestinalis XB6B4 (×3)	
291543183		Ruminococcus bromii L2-63 (×3)	

There were several enzymes of interest coded by *Lactobacillus acidophilus* alone, many involving methionine and cysteine metabolism. Arginase produces ornithine from arginine, a precursor to glutamate. Mercury (II) reductase is included, because sulfur metabolism is involved with detoxifying toxic heavy metals, such as mercury. NADH peroxidase is an antioxidant, and the enzymes associated with methionine metabolism are ultimately associated with glutathione formation, another antioxidant. See Table 8 for these results.

**Table 8:** Enzymes of interest coded by Lactobacillus acidophilus: Query 1.

2.1.1.10	Homocysteine *S*-methyltransferase.	*S*-methyl-l-methionine + l-homocysteine = 2 l-methionine.
2.1.1.14	5-methyltetrahydropteroyltriglutamate homocysteine *S*-methyltransferase.	5-methyltetrahydropteroyltri-l-glutamate + l-homocysteine = tetrahydropteroyltri-l-glutamate + l-methionine.
3.5.3.1	Arginase.	l-arginine + H_2_O = l-ornithine + urea.
4.2.1.22	Cystathionine beta-synthase.	l-serine + l-homocysteine = l-cystathionine + H_2_O.
4.4.1.1	Cystathionine gamma-lyase.	l-cystathionine + H_2_O = l-cysteine + NH_3_ + 2-oxobutanoate.
1.16.1.1	Mercury(II) reductase.	Hg + NADP^+^ + H^+^ = Hg^2+^ + NADPH.
2.1.1.176	16S rRNA (cytosine(967)-C(5))-methyltransferase.	*S*-adenosyl-l-methionine + cytosine(967) in 16S rRNA = *S*-adenosyl-l-homocysteine + 5-methylcytosine(967) in 16S rRNA.
1.11.1.1	NADH peroxidase.	NADH + H_2_O_2_ = NAD^+^ + 2 H_2_O.

Other enzymes of interest were antioxidants superoxide reductase (1.15.1.2), coded by *Faecalibacterium prausnitzii*, and glutathione peroxidase (1.11.1.9), coded by *Prevotella ruminicola*. Several others exhibited metabolic pathways associated with glutamate, cysteine and methionine and a few for some amino acids of less interest, such as serine or histidine.

The following (Table 9) are some enzymes of interest from the “autism” microbiome. Interestingly, *Clostridium perfringens* is the only organism studied (among both microbiomes) that codes for glutamate: cysteine ligase and glutathione synthase, the two enzymes needed for glutathione formation.

**Table 9:** Enzymes of interest coded by the “autism” microbiome; Query 1.

110676061	Clostridium perfringens ATCC 13124	4.1.1.50	Adenosylmethionine decarboxylase.	*S*-adenosyl-l-methionine = *S*-adenosyl 3-(methylthio)propylamine + CO_2_.
115252745	Peptoclostridium difficile 630	4.1.1.50	Adenosylmethionine decarboxylase.	*S*-adenosyl-l-methionine = *S*-adenosyl 3-(methylthio)propylamine + CO_2_.
110676061	Clostridium perfringens ATCC 13124	4.1.1.22	Histidine decarboxylase.	l-histidine = histamine + CO_2_.
110676061	Clostridium perfringens ATCC 13124	6.3.2.2	Glutamate–cysteine ligase.	ATP + l-glutamate + l-cysteine = ADP + phosphate + gamma-l-glutamyl-l-cysteine.
110676061	Clostridium perfringens ATCC 13124	6.3.2.3	Glutathione synthase.	ATP + gamma-l-glutamyl-l-cysteine + glycine = ADP + phosphate +glutathione.
219869941	Desulfovibrio desulfuricans ATCC 27774	2.6.1.44	Alanine–glyoxylate transaminase.	l-alanine + glyoxylate = pyruvate + glycine.
219869941	Desulfovibrio desulfuricans ATCC 27774	1.8.99.3	Hydrogen sulfite reductase.	(O_3_S.S.SO_3_)^2−^ + acceptor + 2 H_2_O + OH^−^ = 3 HSO_3_^−^ + reduced acceptor.
115252745	Peptoclostridium difficile 630	4.4.1.11	Methionine gamma-lyase.	l-methionine + H_2_O = methanethiol + NH_3_ + 2-oxobutanoate.
115252745	Peptoclostridium difficile 630	1.8.1.2	Sulfite reductase (NADPH).	H_2_S + 3 NADP^+^ + 3 H_2_O = sulfite + 3 NADPH.
115252745	Peptoclostridium difficile 630	5.4.3.5	D-ornithine 4,5-aminomutase.	d-ornithine = (2*R*,4*S*)-2,4-diaminopentanoate.
115252745	Peptoclostridium difficile 630	5.1.1.12	Ornithine racemase.	l-ornithine = d-ornithine.

It was found that *Desulfovibrio desulfuricans* and *Clostridium difficile* uniquely coded for enzymes that involved the use of thioredoxins, including sarcosine reductase, betaine reductase and glycine reductase.

Queries 2 and 3 had much less novel results in comparison. We will look at these results first, then take a look at why adding new EC number annotations to the files did not change much in terms of the overall results.

Query 2 only resulted in one new meaningful result (Table 10): the conversion of *S*-adenosyl-methionine to *S*-adenosyl-homocysteine, part of the transsulfuration pathways.

**Table 10:** SAM (*S*-adenosyl-methionine) conversion to SAH (*S*-adenosyl-homocysteine).

219869941	Desulfovibrio desulfuricans ATCC 27774	2.1.1.77	Protein-l-isoaspartate (d-aspartate) *O*-methyltransferase	*S*-adenosyl-l-methionine + protein l-isoaspartate = *S*-adenosyl-l-homocysteine + protein l-isoaspartate α-methyl ester.

Query 3 also resulted in only one major meaningful result, as well as two more that are at least noteworthy, involved with cyanide metabolism (Table 11). Of course, cyanide is highly toxic to humans, so the fact that *Clostridium difficile* is the only organism studied that can metabolize it is interesting, but its relevance to the present study is inconclusive. Also of note was that *Bifidobacterium* and *Lactobacillus* of the control microbiome uniquely coded for 2-haloacid dehalogenase, and *Lactobacillus* uniquely coded for haloalkane dehalogenase. Again, upon searching the literature in regards to these results, the interpretation is inconclusive. The relevance to the present study cannot be determined.

**Table 11:** Enzymes of interest coded by the “autism” microbiome in Query 3.

115252745	Peptoclostridium difficile 630	2.8.1.2	3-mercaptopyruvate sulfurtransferase.	3-mercaptopyruvate + cyanide = pyruvate + thiocyanate.
115252745	Peptoclostridium difficile 630	2.8.1.1	Thiosulfate sulfurtransferase.	Thiosulfate + cyanide = sulfite + thiocyanate.
115252745	Peptoclostridium difficile 630	4.4.1.8	Cystathionine beta-lyase.	l-cystathionine + H_2_O = l-homocysteine + NH_3_ + pyruvate.
666001751	Bifidobacterium longum BXY01	3.8.1.9	(*R*)-2-haloacid dehalogenase.	(*R*)-2-haloacid + H_2_O = (*S*)-2-hydroxyacid + halide.
666001751	Bifidobacterium longum BXY01	3.8.1.2	(*S*)-2-haloacid dehalogenase.	(*S*)-2-haloacid + H_2_O = (*R*)-2-hydroxyacid + halide.
666001751	Bifidobacterium longum BXY01	3.8.1.10	2-haloacid dehalogenase (configuration-inverting).	(1) (*S*)-2-haloacid + H_2_O = (*R*)-2-hydroxyacid + halide. (2) (*R*)-2-haloacid + H_2_O = (*S*)-2-hydroxyacid + halide.
488447870	Lactobacillus acidophilus La-14	3.8.1.2	(*S*)-2-haloacid dehalogenase.	(*S*)-2-haloacid + H_2_O = (*R*)-2-hydroxyacid + halide.
488447870	Lactobacillus acidophilus La-14	3.8.1.5	Haloalkane dehalogenase.	1-haloalkane + H_2_O = a primary alcohol + halide.

In order to analyze why some organisms provided more information than others, we compared the number of EC number annotations within each GenBank file to the total CDSs (coding sequences) within each file. For the new organisms added for Queries 2 and 3, we can see that very few annotations were actually added compared to the total CDSs. “Good” coverage seems to lie between 12% and 25% when taking the ratio of EC number annotations to total CDSs, and all five of the newly annotated files fall well short of this mark. However, Bifidobacterium had greatly improved coverage after updating EC number annotations, rising from <4% to >25%. Despite this, very few new results were obtained. Table 12 shows these statistics. This table is included in the Supplemental Material as Table S8 as well. It may seem strange at first that there are so few EC number annotations compared to the number of annotated CDSs, but keep in mind that enzymes are only a fraction of the organisms’ proteome (e.g., non-catalytic proteins, such as inter-membrane proteins), and some of the organisms’ genes code for non-translated RNA transcripts, such as tRNAs. There also seems to be a dearth in annotation of EC numbers in general.

**Table 12:** GenBank file statistic comparisons between the number of EC number annotations and coding sequences (CDSs).

	Original File			Updated EC Annotations				
Organism Name	Total CDSs	Total EC Numbers	%	Total EC Numbers	Total “Multi-EC” Designations	Actual EC Total	%	% Increase
Akkermansia muciniphila	2138	286	13.38	401	34	367	17.17	28.32
Alistipes putredinis	659	92	13.96	110	6	104	15.78	13.04
Alistipes shahii	2563	548	21.38	603	17	586	22.86	6.93
Bacillus subtilis	4140	912	22.03	941	8	933	22.54	2.30
Bacteroides fragilis	4406	366	8.31	377	5	372	8.443	1.64
Bifidobacterium longum	1903	68	3.57	672	189	483	25.38	610.29
Clostridium difficile	3902	1015	26.01	1,035	6	1029	26.37	1.38
Clostridium leptum	602	100	16.61	114	4	110	18.27	10.00
Clostridium perfringens	2878	504	17.51	539	10	529	18.38	4.96
Desulfovibrio desulfuricans	2356	292	12.39	392	32	360	15.28	23.29
Escherichia coli	4967	612	12.32	1542	285	1257	25.31	105.39
Eubacterium rectale	2898	636	21.95	696	16	680	23.46	6.92
Faecalibacterium prausnitzii	2756	586	21.26	641	17	624	22.64	6.48
Lactobacillus acidophilus	1876	486	25.91	585	31	554	29.53	13.99
Methanobrevibacter smithii	1795	414	23.06	454	12	442	24.62	6.76
Odoribacter splanchnicus	3498	515	14.72	603	27	576	16.47	11.84
Prevotella ruminicola	2791	480	17.20	519	12	507	18.17	5.62
Roseburia intestinalis	3630	709	19.53	793	17	776	21.38	9.45
Ruminococcus bromii	1811	467	25.79	496	9	487	26.89	4.28
Weissella koreensis	1335	93	6.97	288	61	227	17	144.09
Bacteroides caccae	3441	0	0.00	76	19	57	1.656	
Bacteroides thetaiotaomicron	4787	0	0.00	413	125	288	6.016	
Bacteroides vulgatus	4065	0	0.00	267	80	187	4.6	
clostridium bolteae	5830	0	0.00	737	227	510	8.748	
Sutterella wadsworthensis	2433	0	0.00	136	39	97	3.987	

## 4. Discussion

The present study successfully identified several key enzymes associated with autism spectrum disorders using a bioinformatics data mining approach, by comparing the metabolomes of two distinct microbiomes. We must compare the results to published experimental data in order to evaluate the impact of this study, such as those found in [29,30,31,32,33].

We expected to find key biomarkers unique to *Desulfovibrio* and *Clostridia* species indicative of autism, but instead, the key was based on identifying enzymes that were missing from these organisms; hence, a decreased abundance of such enzymes could be used as diagnostic biomarkers for autism. Several different amino acids have previously been reported to be in lower abundance in autistic individuals, but in this study, the amino acid that stood out the most was glutamate. Several organisms in the “control” microbiome coded for enzymes associated with glutamate metabolism, but were curiously missing from the “autism” microbiome. Glutamate is an integral part of glutathione, a tripeptide made of glutamate, cysteine and glycine. It was known before that glutathione was down-regulated in autism, but researchers focused their attention on an inhibition of cysteine metabolism to be the culprit.

Upon closer inspection of glutamate’s association with autism, we find that there is a hypothesis that does not involve oxidative stress at all. Glutamate is the human body’s major excitatory neurotransmitter, which works in opposition to gamma-aminobutyric acid (GABA) [34,35]. Contrary to the Ming *et al.* study [29], these studies, as well as Adams *et al.* [31] reported higher quantities of glutamate *vs.* neurotypical controls. Tevarst van Elst *et al.* [34] actually reviewed two different hypotheses: that a hypoglutamatergic condition is related to autism and also that a hyperglutamatergic condition is related to autism. Regardless of which one it is, the authors theorize that an imbalance in the neurological excitation/inhibition chemical signaling in the central nervous system is thought to be associated with autism. Furthermore, glutamate decarboxylase is the enzyme responsible for converting glutamate into GABA. Loss of the gene coding for GABA in host neurons has been shown to lead to symptoms characteristic of autism [36]. Going back to the results of our software (*i.e.*, Query 2), we found that glutamate decarboxylase is expressed by organisms from both the control and autism groups. Therefore, what then is the association of our research to the hypotheses on glutamate and autism? That remains to be determined, but we must not ignore the fact that we might not have heard of this field of research had not our software pointed in that direction. Even with limited data availability, it seems that our software can be useful in developing new hypotheses and revealing literature that was previously unknown to us.

After completing our analysis, we came across another study where *Akkermansia muciniphila* was found in decreased amounts in individuals with autism [37]. According to the authors, *Akkermansia* is integral to the host’s gut health; the gut mucus layer is reduced as *Akkermansia* composition within the gut is depleted. This contradicts the Finegold *et al.* pyrosequencing study. In light of these two conflicting studies, it seems that we may have been correct in assuming that the involvement of *Akkermansia* in autism is questionable at best. We still included it in the study, because theoretically, it should serve as a basis for comparison. If we think that certain *Clostridia* species and sulfate reducers are indicative of autism, then we may be biased towards results that agree with that assumption. If we include a questionable case, such as *Akkermansia*,in the “autism” group analysis, then we may better be able to rule out false positives. Regarding this inclusion, no significant results were found in Queries 1 or 2, leading to the exclusion of *Akkermansia* from Query 3. Query 3 had quite similar results as Queries 1 and 2, which lends credence to the idea that those organisms still included in the “autism” group for Query 3 are more likely to be involved in autism pathogenesis.

Hydrogen sulfide has been cited as a toxic metabolite produced by *Desulfovibrio* species [10], but data on this metabolite were inconclusive. It was found that *Desulfovibrio* is not the only organism (in either microbiome) that can metabolize hydrogen sulfide. What we did notice is that *Clostridium difficile* codes for enzymes that metabolize alternative pathways for ornithine and methionine, precursors to glutamate and cysteine, respectively, with the enzymes ornithine racemase, d-ornithine 4,5-aminomutase, adenosylmethionine decarboxylase and methionine gamma-lyase. Nearly every organism studied codes for enzymes using ferredoxins and thioredoxins, alternative antioxidants to glutathione, which also use a sulfhydryl electron acceptor for reducing power. It is possible that thioredoxins were found in much higher abundance in autistic individuals [28], because thioredoxins had to take over from glutathione as the body’s dominant antioxidant. However, if glutathione is usually dominant, then there must be something not as chemically favorable associated with ferredoxins and thioredoxins or perhaps they are just not sufficient to fill in the antioxidative gap that glutathione usually fills. That *Clostridium difficile* can uniquely use ornithine and methionine for other purposes other than glutathione formation provides evidence for this hypothesis. Interestingly, the final two catalytic steps in glutathione formation are uniquely coded by *Clostridium perfringens*, part of the “autism” microbiome. However, many of the precursor steps are uniquely coded by *Lactobacillus acidophilus*, part of the “control” microbiome. Glutathione is still existent in autistic individuals, so perhaps *Clostridium perfringens* allows for some extra utility in glutathione formation; or maybe this is just coincidence.

Quite remarkable was the fact that nearly every reaction of the transsulfuration and transmethylation metabolic pathways was coded by the organisms studied, but that each microbiome (“control” and “autism”) could not express the pathways in their entirety alone. This suggests some sort of interdependence between the two microbiomes on completing these pathways. The control microbiome supplies glutamate metabolism, and cysteine and glutathione formation is split between the two groups of organisms. Based on this information, it seems that organisms, such as abnormal *Clostridia* species and *Desulfovibrio*, may be products of the sulfur metabolic deficiency found in so many autistic individuals and, therefore, may be essential in filling a unique metabolic niche. However, because of the known toxic byproducts of these organisms, they may still be causing gastrointestinal inflammation and subsequent neuro-inflammation associated with “leaky gut syndrome”, leading to regressive autism [15,31]. Therefore, even though these organisms may be causing some major ill effects, the matter becomes complicated by the necessity of their presence in the host. The sulfur metabolic deficiencies and lack of proper anti-oxidation therefore seem to lie at the heart of the problems that lead to regressive autism. If treatment is possible, it would therefore be more likely to entail dietary supplements that restore the metabolic deficiencies to normal conditions [13], rather than eradicating the proposed offending organisms from the host’s gut. James *et al.* [13] reported some improvements in symptoms with specific dietary supplements, and a study by Adams *et al.* [38] provides some more evidence of this. The Adams *et al.* study included adult subjects with autism, as well.

It is important to note that there were some crucial limitations to this study. Besides the obvious, that this study is purely computational, the dependence on having annotation data for EC numbers greatly limits the scope of the study. When the first query to the web application was completed and analyzed, it was thought that including more organisms of interest (with newly annotated EC numbers) would greatly strengthen the study. This, in fact, hardly had any impact at all on the initial study. These files had so few annotations, that they did not give good representations of the metabolome of these organisms. Thus, we can only surmise that there may be other more meaningful results possible with better data. It is possible that this study could be improved by creating new gene annotations altogether by using the publically available BLAST tool for sequence alignment of microbial genomic DNA and then annotating these genes with translated gene products and EC numbers. Also of note is that the two *Clostridium* species studied most deeply in this context (*C. perfringens* and *C. difficile*) have not, to our knowledge, been specified as part of the “autism” microbiome, but they happened to be two of the most widely available and comprehensive genomes of *Clostridia* that could appear in the human gut. Therefore, they were chosen as “representatives” of the *Clostridium* genus, and thus, associated results should be interpreted with scrutiny. It appears that this type of study has not been attempted previously, yielding mixed results. This provides substantial evidence that the present approach is novel and may be a step in a new, potentially useful direction, but its novelty severely limits its impact, because of a lack of resources. It is surmised that as data on microbial genomics and data on gut bacteria associated with autism patients improves, so too will the utility of our novel software and our comparative modeling approach. Future work could include full *de novo* genomic sequencing of gut microbes and validation through next-generation sequencing devices, followed by comprehensive annotation with accurate EC numbers. On NCBI’s server, there is a noted difference in genomic comprehensiveness between microorganisms, where key gut microbes are covered definitively less than model organisms, such as *Escherichia coli* or *Bacillus subtilis*. We need to be able to have more studies on gut microbes in their natural habitat and their interactions with host cells in order to have more experimental data to fine-tune our metabolomics model. It also remains to be determined what would be the best way of evaluating data significance from this type of study.

Nevertheless, we still believe that substantial results came about from this study. We have not seen this type of comparative analysis conducted with autism in mind before, and we are proud to say that our computational approach was successful, given some of its parallels to previous experimental studies. Some experimental studies were not able to be validated with this approach, which is to be expected. As stated earlier, this software was designed with the intent to be used for many other applications, besides autism, hence making the software publically available online. Many other human illnesses and conditions are thought to be associated with the human gut microbiome [5], and we believe that these applications might benefit from our approach, as well. Microbiomes in other environments may also benefit; much potential could be lost if the software’s use were limited to only the human gut.

## Supplemental Materials

Supplementary materials can be found at http://www.mdpi.com/1422-0067/16/04/8949/s1.
